# Electroacupuncture to treat with Overactive Bladder in Postmenopausal Women: study protocol for a multicenter, randomized, controlled, parallel clinical trial

**DOI:** 10.1186/s13063-018-2715-3

**Published:** 2018-09-15

**Authors:** Eun-Young Nam, Ju-Yeon Park, Junyoung Jo, Seung-Hyun Jung, Chi-Yeon Lim, Dong-Il Kim

**Affiliations:** 1 Department of Obstetrics & Gynecology, College of Korean Medicine, Gachon University Dongincheon Gil Korean Medicine Hospital, 21 Keunumul-ro, Jung-gu, Incheon, 22318 Republic of Korea; 2 Research institute of Korean Medicine, College of Korean Medicine, Dongguk University, 32 Dongguk-ro, Ilsandong-gu, Goyang-si, Gyeonggi-do 10326 Republic of Korea; 3 Department of Obstetrics and Gynecology Conmaul Hospital of Korean Medicine 110 Seochojungang-ro, Seocho-gu Seoul, 06634 Republic of Korea; 4 Department of Internal Medicine, College of Korean Medicine, Dongguk University Bundang Oriental Hospital, 268 Buljeong-ro, Bundang-gu, Seongnam-si, Gyeonggi-do 13601 Republic of Korea; 5 Department of Biostatistics, College of Medicine, Dongguk University, 32 Dongguk-ro, Ilsandong-gu, Goyang-si, Gyeonggi-do 10326 Republic of Korea; 6 Department of Obstetrics & Gynecology, College of Korean Medicine, Dongguk University Korean Medicine Hospital, 27 Dongguk-ro, Ilsandong-gu, Goyang-si, Gyeonggi-do 10326 Republic of Korea

**Keywords:** Overactive bladder, Menopause, Postmenopausal overactive bladder, Electroacupuncture

## Abstract

**Background:**

Electroacupuncture has been used for treatment in patients with overactive bladder. This study was conducted to evaluate the efficacy and safety of electroacupuncture for treating overactive bladder of postmenopausal women.

**Methods/design:**

This is a multicenter, randomized controlled, parallel clinical trial. Two hundred ninety participants with overactive bladder syndrome will be recruited from Dongguk University Bundang Oriental Hospital and Cheonan Korean Medicine Hospital of Daejeon University and randomly allocated into one of two groups in a 1:1 ratio. One group will receive electroacupuncture (EA) and the other acupuncture (AC). The allocation will be concealed from both participants and assessors. The study period will be about 10 weeks, including 6 weeks of electroacupuncture or acupuncture treatment and a four week follow-up period. Both EA group and AT group will undergo acupuncture at 7 fixed points, and the EA group will undergo electronic stimulation at 6 points. The primary outcome will be the average number of micturitions per 24 h based on a 3-day bladder diary. The secondary outcome will comprise the 3-day bladder diary, the overactive bladder symptom score and the results of the King’s health questionnaire.

**Discussion:**

The results of this trial will provide information regarding the efficacy and safety of electroacupuncture for treating overactive bladder in postmenopausal women.

**Trial registration:**

ClinicalTrials.gov, NCT03260907. Registered on 24 August 2017.

**Electronic supplementary material:**

The online version of this article (10.1186/s13063-018-2715-3) contains supplementary material, which is available to authorized users.

## Background

Overactive bladder (OAB) is a highly prevalent disorder that has a negative impact on quality of life [1, 2]. OAB is defined as urinary urgency, usually accompanied by frequency and nocturia, with or without urgent urinary incontinence, in the absence of urinary tract infection (UTI) or other obvious pathology [3–5]. In patients with OAB, frequency (85%) is the most commonly reported symptom, followed by urgency (54%) and urge incontinence (36%) [6].

The condition is more common in women and in people over 40 years of age [2, 3]. The prevalence of OAB increases with age and duration of menopause [6]. The overall prevalence of OAB in Korean women has been shown to be 14.3%, while it was 18.4% for those aged over 40 [7, 8].

Acupuncture, which is one of the primary treatments utilized traditional Korean medicine (TKM), involves various stimulation methods such as electrical devices, moxibustion, pharmacopuncture, and thread-embedding therapy to stimulate acupoints, for controlling bladder function at the lower abdomen and lumbar-sacral area [8]. Among these methods, electroacupuncture is a typical and common treatment for OAB in TKM, which is effective at suppressing excessive contractions and activities of the detrusor muscle, improving bladder compliance, maintaining normal urination, and improving pathological changes in bladder tissue [9, 10]. Mechanical stimulation is supposed to send signals to the spinal cord via the sensory ganglia and interneurons, thereby regulating the activity of motor neurons in the brain stem that control autonomic functions, including urinary activity [11, 12]. However, the efficacy and safety of electroacupuncture are still controversial.

Recent clinical studies have evaluated the efficacy electric stimulation for treating OAB [13] However, a randomized controlled trial (RCT) for validation of the comparative efficacy and safety of acupuncture with electric stimulation as concurrent treatment has not yet been undertaken.

This study will evaluate the efficacy and safety of electroacupuncture for treating OAB of menopausal women.

## Methods/design

### Study design

**Fig. 1:**
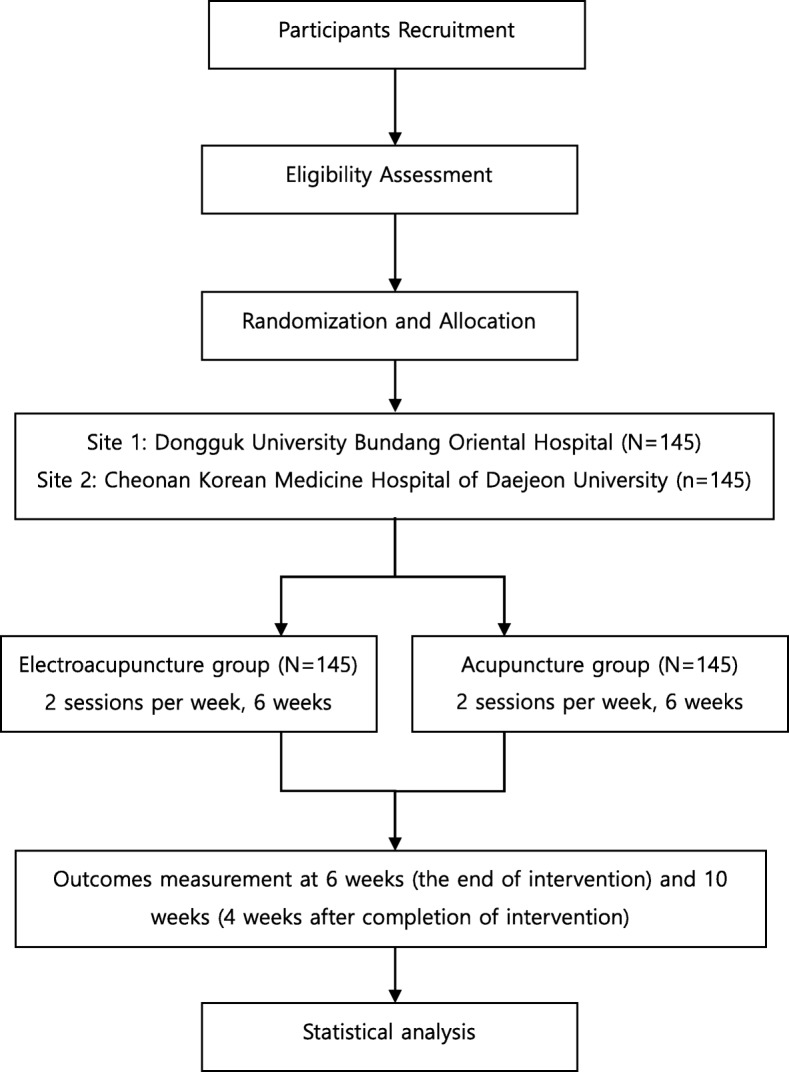
Flowchart of the study process

This is a multicenter, randomized controlled, parallel clinical trial. The study protocol conforms to the Consolidated Standards of Reporting Trials (CONSORT) [14] and Standards for Reporting Interventions in Clinical Trials of Acupuncture (STRICTA) [15] guidelines. The study process is illustrated in Fig. 1. The institutional review board of Dongguk University Bundang Oriental Medical Hospital approved the study (2017–0008). The protocol has been registered in ClinicalTrial.gov (NCT03260907).

Participants will be recruited through advertisements in outdoor areas as well as on hospital websites. Two hundred ninety participants with overactive bladder syndrome will be randomly allocated to one of two in a 1:1 ratio. One group will be the electroacupuncture (EA) group, and the other will be the acupuncture (AC) group. The study period will be about 10 weeks, including 6 weeks of electroacupuncture or acupuncture treatment and a 4 week follow-up period. The schedules for enrollment, intervention, and assessments is shown in Table 1. The SPIRIT checklist is provided in Additional file 1.

**Table 1:** Schedule to be used for enrolment, intervention, and assessments. OABSS, overactive bladder symptom score; KHQ, King’s health questionnaire

	Study period													
	Screening	Treatment												Follow-up
Week	0	1		2		3		4		5		6		10
Visit	1	2	3	4	5	6	7	8	9	10	11	12	13	14
Enrollment:														
Informed consent	X													
Demographic characteristics	X													
Medical history	X													
Vital signs	X	X	X	X	X	X	X	X	X	X	X	X	X	X
Physical examination	X												X	
Urinalysis	X												X	
Inclusion/Exclusion criteria	X													
Random allocation		X												
Intervention:														
Acupuncture treatment		X	X	X	X	X	X	X	X	X	X	X	X	
Assessments:														
3-day bladder diary		X											X	X
OABSS	X												X	X
KHQ	X												X	X
Adverse events		X	X	X	X	X	X	X	X	X	X	X	X	X

### Eligibility criteria

Two hundred ninety patients with OAB will be recruited from two hospitals in Korea: Dongguk University Bundang Oriental Hospital in Bundang, and Cheonan Korean Medicine Hospital of Daejeon University in Cheonan. After obtaining informed consent from the patients or their representatives, we will screen them according to the eligibility criteria for enrollment in the study.

### Inclusion criteria

The inclusion criteria for the study will be as follows: (1) women over 40 years of age without the possibility of pregnancy; (2) those who have a history of amenorrhea for at least 1 year and have no previous history of hormone replacement therapy for the last 6 months; (3) those who have symptoms of urinary frequency and urgency lasting more than 3 months; (4) those who fit the diagnostic criteria for OAB, with a total score of more than 3 points on the Korean version of the Overactive Bladder Symptom Score (OABSS); (5) those who have an average urinary frequency of more than eight times per day and urgency defined by the urgency rating scale (URS) on the bladder diary of more than 2 points and/or urgent urinary incontinence (UUI) on the 3-day bladder diary during a 1-week screening period; (6) those who agree to this clinical study after sufficient explanation.

### Exclusion criteria

The exclusion criteria for the study are as follows: (1) diagnosed with UTI by urine examination; (2) having stress urinary incontinence without symptoms of OAB; (3) suspected of having voiding dysfunction induced by neurological damage; (4) a medical history of cystocele, uterine prolapse or similar condition; (5) a medical history of obstructive uropathy such as urinary stones and urinary tumors; (6) a surgical history of urethra or bladder; (7) a medical history of malignant tumors of the urinary tract; (8) a medical history of neurological disease or psychiatric illness; (9) an artificial cardiac pacemaker or implantable cardioverter defibrillator in the chest; (10) having experienced a hypersensitivity reaction after an acupuncture treatment, or showing any other contraindications; (11) having participated in another clinical trial within the past three months; (12) having taken therapeutic drugs that may affect bladder function within one month of the start of this study; (13) having inadequate literacy to complete the study documents.

### Dropout criteria

Patients will be removed from the study if they are unwilling to continue their participation in the study. Patients who fail or anticipate that they might fail to attend at least 10 sessions will also be dropped from the study.

### Randomization, allocation concealment and blinding

A table of random numbers will be produced according to sample size using the statistical software SAS (version 9.4; SAS Institute Inc., Cary, NC, USA), and sealed in opaque envelopes. The envelopes will be delivered to each research center and stored in a double-locked cabinet. The practitioner will open the corresponding envelope and allocate the participants who meet the eligibility criteria after signing an informed consent form. Participants will be randomly allocated to the electroacupuncture (EA) group or the acupuncture (AC) group at a ratio of 1:1. After the practitioner checks the group allocation for the subject, the envelope will be stored again in a separate, double-locked cabinet.

Randomization will be stratified by centers and is conducted with concealment of the randomization list.

Practitioners cannot be blinded, and they will be excluded from the assessment procedure. Outcomes will be assessed by researchers who are not involved in the intervention procedure.

### Interventions

The intervention treatment period includes 12 sessions over 6 weeks. The participants will visit the research center twice a week through the 6 week period. Licensed Korean medical doctors who have at least 1 year of clinical experience will perform the intervention. Detailed information regarding the schedule of the enrolment, interventions, and assessments is provided in Additional file 1 and Table 1.

For the EA and AC groups, disposable, sterilized, filiform needles (diameter 0.25 mm, length 40 mm; Dong-Bang Acupuncture Inc., Seoul, Korea) will be inserted into the skin at the following acupoints: unilaterally at CV3, CV4, and GV20, bilaterally at KI3 and SP6. When inserting at CV3, the needling direction will be toward CV2. The needles will be inserted to a depth of between 5 and 30 mm, and the acupuncture needles will be manipulated to achieve de qi and retained for 20 min.

In the EA group, electrical stimulation will be performed simultaneously during the retaining period. An electric acupuncture device (CellMac STN-110, Stratek Co., Republic of Korea) will be connected to the CV3-CV4 and KI3-SP6 acupoints and deliver stimulation with a 2 Hz frequency, asymmetric bimodal pulse, continuous wave mode, and a maximum intensity below the threshold (7.6–13.9 mA). Electrical stimulation will be delivered at an intensity that the participant can notice but will still feel comfortable with.

### Prohibited and permitted concomitant treatment

Participants will be prohibited from receiving any OAB treatment outside of the interventions of this trial during the 6-week treatment period. If a patient start medication for OAB during the treatment period, they will be withdrawn from the trial. All new treatments started after the beginning of the trial and concomitant medications to treat medical conditions unrelated to OAB will be recorded on the Case Report Form (CRF).

### Outcomes

Although it is impossible to ensure blinding for intervention conductor and the participants, we will ensure blinding for the outcome measurements analyses by independent assessors unaware of group allocation. The following outcomes will be assessed by independent assessors blinded to the allocation. Efficacy will be assessed by the 3-day bladder diary, the OABSS and the KHQ.

#### Primary outcome

The primary outcome measurement of this study is the average number of micturitions per 24 h based on the 3-day bladder diary at the end of the treatment period (week 6).

Participants will complete a 3-day bladder diary before and after treatment (week 6), and the follow-up period (week 10). Entries will be recorded in micturition diaries for 3 days before each follow up visit. Participants will record the time of every micturition and rate the intensity of urgency using the five-point URS (1 = no urgency, 2 = mild urgency, 3 = moderate urgency, 4 = severe urgency, 5 = UUI).

#### Secondary outcome

Secondary outcome measurements will include the number of micturitions in the 3-day bladder diary at the end of the treatment period (week 6) and the follow-up period (week 10). The same version of the 3-day bladder diary will be applied again. This includes daytime micturitions per 24 h, nocturnal micturitions per 24 h, total count of urgency (sum of urgency episodes defined as URS ≥ 3 for three days), total urgency score (sum of urgency score for three days), and total count of UUI (sum of UUI episodes for three days).

In addition, the OABSS and the KHQ will be measured as secondary outcomes. Participants will complete the Korean version of the OABSS and the KHQ at baseline, the end of the treatment period (week 6), and the follow-up period (week 10).

The OABSS is an assessment tool for OAB symptoms that was developed and validated in Japanese populations in 2006 [16]. The OABSS consists of four questions regarding OAB symptoms; daytime frequency, nocturia, urgency, and UUI. The sum of the four scores runs between 0 and 15. The diagnostic criteria for OAB is a total OABSS of ≥3 with an urgency score for question 3 of ≥2 [17].

The KHQ is an assessment tool for quality of life data of OAB symptoms that was developed by Kelleher and colleagues [18, 19]. The KHQ is a urine questionnaire that can assess the severity of urination symptoms and evaluate the impact of urination symptoms on quality of life. This questionnaire is known to have reliability and validity for measuring the quality of life of patients with incontinence [20]. The 10 domains from the KHQ evaluated are general health perception, incontinence impact, role limitations, physical limitations, social limitations, personal relationships, emotions, sleep/energy, severity measures, and symptom severity.

### Safety assessment

All participants will be monitored for adverse events during the study. Adverse events (AEs) indicate undesirable and unintentional signs, symptoms, or diseases that develop after intervention during the period of a clinical trial. They do not necessarily have a causal relationship with the relevant intervention. Investigators will check participants’ vital signs and examine for manifestation of AEs at each visit. Investigators will also conduct physical examinations and laboratory tests, including evaluation of urinalysis (specific gravity, nitrite, pH, protein, glucose, ketones, urobilinogen, bilirubin, blood, white blood cells, urine HCG) at the screening as well as at the end of treatment period (week 6). The outcome assessors will judge the severity (none, mild, moderate, or severe), seriousness, and the correlation between AEs and intervention (definitely related, appears to be related, possibly related, appears to be unrelated, definitely not related, or unclear).

### Sample size

The primary outcome of this study will be the average number of micturitions per 24 h after the treatment. Based on a previous study [21], we estimated that the mean difference in the pre- and post-treatment change in the average number of micturitions per 24 h scores between the EA and AC group will be 0.8 and the standard deviation will be 2.1. With a 5% significance level and 80% power, the needed sample size was calculated to be 116 per group. Considering a 20% drop-out rate, 145 participants are required per group. In conclusion, a total of 290 participants are needed in this trial.

### Statistical analysis

We will analyze results primarily based on the principle of intention-to-treat (ITT) and subordinately based on the per-protocol (PP) principle. The ITT analysis will include any participants who are randomly allocated and have at least one EA or AC session. The PP analysis will cover only patients who have completed at least 10 sessions of EA or AC treatments with no use of medication or medical device of UTI or OAB, and have no serious violation. The primary and secondary continuous outcomes will be compared between randomized groups using ANCOVA models that includes baseline measurement as covariates.

The categorical variables will be analyzed by the Pearson χ^2^ test or Fisher’s exact test. If there is a significant difference between groups in terms of baseline characteristics, analysis of covariance (ANCOVA) or logistic regression for further evaluation will be performed. When it is necessary to control important variables that may affect the final evaluation, stratification analysis (Cochran-Mantel-Haenszel method, etc.) will be applied.

## Discussion

This will be a multicenter, randomized controlled, parallel clinical study designed to evaluate the efficacy and safety of electroacupuncture treatment of postmenopausal women with OAB, as well as to clarify the effects of electro stimulation of acupuncture on urinary activity. According to recent studies, insufficient evidence exists regarding the efficacy of EA in patients with OAB. Electro-stimulation (ES) is likely to improve OAB symptoms or OAB-related quality of life more than no active treatment, but it is uncertain whether ES is better than other treatments, such as pelvic floor muscle training (PFMT) or drug therapy [17]. However, percutaneous posterior tibial nerve stimulation (PTNS), which is a kind of electroacupuncture at SP 6, is generally examined as effective treatment option of OAB [22, 23]. Moreover, the studies suggest that electroacupuncture compares favorably to drug therapy with a viable long term effect [24–26].

Overactive bladder is an asymptomatic diagnosis that has been defined as comprising symptoms of frequency of more than eight micturitions per 24 h, urgency and urge incontinence, occurring singly or in combination and not explained by metabolic conditions such as diabetes or local pathological factors such as urinary tract infection (UTI), stones, or interstitial cystitis. Patients with an overactive bladder include those with and without a possible neurological cause for their symptoms [27]. Overactive bladder is a chronic condition defined urodynamically as detrusor overactivity that is characterized by involuntary bladder contractions during the filling phase of the micturition cycle [28]. The involuntary contractions result in reduced functional bladder capacity and unpredictable, troublesome symptoms [7]. In clinics, the diagnosis of OAB is based on urinary symptoms, detailed history, overactive bladder symptom score (OABSS), bladder diary, and physical examination. For clinical examination, urinalysis is performed to rule out UTI, and ultrasonography is considered if a female genital tumor is suspected.

The investigators will target postmenopausal women with OAB. After treatment in two groups (electroacupuncture or acupuncture), the investigators will compare urinary symptoms assessed based on the 3-day bladder diary and overactive bladder symptom score (OABSS) as well as the quality of life assessed by King’s Health Questionnaire (KHQ) and evaluate the safety of abnormal reaction during the treatment.

In this study, the EA group and AT group will undergo acupuncture at 7 fixed points, and the EA group will include electronic stimulation at 6 points. The efficacy of electric stimulation of acupuncture will be compared to that of acupuncture without any stimulation.

The 7 acupoints were identified based on the guidelines published by the World Health Organization. In the abdomen region, CV 3 and CV 4 have been reported to possibly have the ability to recover bladder dysfunction in literature pertaining to traditional Korean medicine, and were shown to have clinical effectiveness for overactive bladder in previous studies [29, 30]. These acupoints are considered to have a possible mechanism in which acupuncture stimulation directly increases the excitability of the pelvic nerve, which consequently innervates the detrusor muscle [9, 10].

The period required for electroacupuncture treatment of OAB is controversial. According to RCTs from Korea and China, about 12–24 weeks are needed to recover and stabilize bladder function with 20–30 or more sessions of regular and long-term electroacupuncture or acupuncture [9], whereas trials lasting 4–12 weeks with 4–24 or more sessions of PTNS were required in a previous systematic review [17]. Based on previous existing studies and actual clinical practices, this study will employ 12 treatment sessions over 6 weeks.

This study is expected to be the first well-designed RCT to determine the effects of adding electro stimulation to acupuncture treatment on OAB. At the end of this project, the findings will provide the clinical evidence of the efficacy and safety of EA as a treatment for OAB in postmenopausal women.

### Dissemination policy

We will report the final data to the Ministry of Health & Welfare through the Korea Health Industry Development Institute. We will also publish the results after study completion.

### Trial status

This trial is proposed. Enrollment and trial procedures are expected to be complete by the end of Dec 2019.

## Additional file

Additional file 1: SPIRIT 2013 Checklist: Recommended items to address in a clinical trial protocol and related documents*. (DOC 130 kb)

## Abbreviations

AC: Acupuncture
AEs: Adverse events
CRF: Case report form
EA: Electroacupuncture
IRB: Institutional review board
ITT: Intention-to-treat
KHQ: Korean version of King’s Health Questionnaire
OAB: Overactive bladder
OABSS: Overactive bladder symptom score
PP: Per Protocol
PTNS: percutaneous tibial nerve stimulation
RCT: Randomized controlled trial
SPIRIT: Standard Protocol Items: Recommendations for Interventional Trials
STRICTA: Standards for Reporting Interventions in Clinical Trials of Acupuncture
TKM: Traditional Korean medicine
UTI: Urinary tract infection
UUI: Urge urinary incontinence
